# From loose sand to sandstone: An experimental approach on early calcite precipitation in sands of siliciclastic and mixed carbonate-siliciclastic composition

**DOI:** 10.1371/journal.pone.0312479

**Published:** 2024-10-23

**Authors:** Meike Janssen, Luca Caracciolo, Linda M. Bonnell, Robert H. Lander, Philipp Adelhardt, Lara Moldenhauer, Axel Munnecke, Robert van Geldern, Harald Stollhofen

**Affiliations:** 1 GeoZentrum Nordbayern, Department of Geography and Geosciences, Friedrich-Alexander-Universität Erlangen-Nürnberg, Erlangen, Germany; 2 Geocosm LLC, Durango, Colorado, United States of America; Soran University, IRAQ

## Abstract

Artificially cemented sandstones were produced to assess the impact of detrital texture and composition on the precipitation and distribution of early calcite cement, and cement-related degradation in porosity. To simulate early-calcite cementation, loose sediment of variable composition (siliciclastic and calcareous) and grain size was exposed to a calcite supersaturated solution for 35 to 58 days at 23°C. Identification and distribution of the newly precipitated crystals was performed with high resolution 2D optical and scanning electron microscopy. The experimental results show the precipitation of grain-coating, pore-bridging and pore-filling granular calcite cement with up to 100 μm crystal size. Despite a positive correlation between the amount of detrital carbonate grains and calcite crystals, calcite cement does not preferentially nucleate on bioclast surfaces, irrespectively of their favourable mineralogy. Siliciclastic grains show high calcite cement coverage with altered feldspar, particularly plagioclase, displaying coverage of 94.3%. Grain size variations within the sand packs have influence on the precipitation pattern of calcite with coarse-grained layers (500–710 μm) showing minor calcite cementation (6.3%), while medium- (250–500 μm) to fine-grained layers (125–250 μm) comprise average calcite cement contents of 16.3% and 28.2%, respectively. The findings of this study enhance our knowledge regarding the precipitation processes of calcite in porous material with heterogeneous reacting mineral phases, shapes and pore connectivity.

## Introduction

Calcite occurs as a common cement phase in sandstones with widespread, but heterogeneous distribution, often occurring as lenses or laterally extensive layers [1–4]. The frequent occurrence and development of calcite cement at any diagenetic stage and in various environments can strongly affect reservoir quality and reduce porosity and pathways for fluid migration [3, 5, 6]. Furthermore, depleted hydrocarbon reservoirs have the potential to serve as carbon dioxide storage sites [7], which also requires accounting for possible reactions with carbonate minerals as they are targeted as favourable mineralogical CO_2_ sinks due to their stability and rapid precipitation kinetics [8–11]. In geothermal systems, the production of high salinity and gas-rich thermal water as well as temperature and pressure drops promote diverse mineral precipitation (scaling) in wells or pipes [12] with carbonate scaling being particularly common in low- to moderate enthalpy systems, like those found in Central Europe [12]. At shallower depths and lower temperatures such aspects are also relevant for the understanding of reservoir heterogeneities in Aquifer Thermal Storage (ATES) and their sustainable use [13]. Examples for geotechnical aspects related to carbonate mineral formation are soil stabilization and construction restoration [14, 15]. Therefore, understanding the nature of calcite precipitation and reactions with CO_2_ or injection fluids under different environmental conditions is highly important both, for estimating reservoir fluid-flow and geomechanical properties and for predicting potential fluid-rock interactions.

Different methods have been described for synthetic calcite precipitation in porous material, including methods utilizing urea hydrolysis, either by urea-producing bacteria (microbial induced, MICP) or the direct use of urease enzymes (enzyme-induced, EICP), to initiate calcite precipitation [15–17]. Other experiments use NaCO_3_ or NaHCO_3_ together with CaCl_2_ as mixing agents for injection solutions [18–20]. Molenaar and Venmans [21] implemented an experiment to simulate natural subtidal cementation processes, resulting in precipitation of continuous fringes of rhombohedral calcite crystals on grains of carbonate composition. Although several experimental approaches have been made to simulate carbonate cementation and calcite reaction kinetics have received great attention during the last decades, the complex interplay of the several controlling processes on calcite precipitation are difficult to constrain and simulate through time [22–24], and the upscaling from pore to reservoir scale remains challenging [25, 26].

The objective of this study is to simulate natural early calcite cementation in sandstone, and cement-related degradation in porosity and permeability. Therefore, flow-through experiments at core scale with sand packs of different detrital composition and texture are conducted to investigate the precipitation processes and distribution of calcite cement. High-resolution 2D optical and scanning electron microscopy were used to track micron scale features.

## Thermodynamic and kinetic concepts of carbonate precipitation

In chemical thermodynamics, the dissolution or precipitation of a solid phase, like calcite, is controlled by the saturation of the solution. In supersaturated conditions there is a drive for the system to reduce the elevated activities of aqueous components to equilibrium values by precipitating a mineral phase. The saturation state Ω, is defined as the ratio of the actual ion product of the solution to the expected ion product at solution equilibrium, or the saturation index (SI):

$$\Omega =\frac{\left[{Ca}^{2+}\right]\;x[{{CO}_{3}}^{2-}]}{K_{sp}}$$ (1)

$$SI=\text{log}\;\Omega =\text{log}\;\left(\frac{\left[{Ca}^{2+}\right]\;x[{{CO}_{3}}^{2-}]}{K_{sp}}\right)$$ (2)

with *K*_*sp*_ being the calcite solubility product, defined as:

$$K_{sp}=[{{Ca}^{2+}]}_{sat}*\;{{{[CO}_{3}}^{2-}]}_{sat}$$ (3)

If Ω > 1 (or equally SI > 0), the solution is supersaturated and will precipitate solid calcium carbonate from the solution to achieve equilibrium (Ω = 1, SI = 0) again. If Ω < 1 (SI < 0), the solution is undersaturated and will dissolve solid calcium carbonate to increase its ion product [27]. Considering sandstone diagenesis, the solubility of carbonate minerals is a function of *p*CO_2_, temperature and formation water chemistry [23, 24]. At constant *p*CO_2_, carbonate minerals show a retrograde solubility trend with increasing temperature [24, 28]. As shown by equation (3), the solubility of calcium carbonate is determined by the carbonate ion concentration, which is dependent on the pH and influenced by changing carbon dioxide concentrations [29]. The composition of intruding waters into the pore space also affects the precipitation and composition of carbonate minerals and is not only determined by external factors, but also by the dissolution of solid mineral phases within the sediment [30].

The process of calcite precipitation can be divided into nucleation and crystal growth. The formation of a crystal from solution initially requires a decrease in Gibbs free energy with the point of minimum free energy being the state of thermodynamic equilibrium and nucleation occurring at the maximum in free energy [27]. The generation of a new phase, like calcite, starts with the formation of small precursor nuclei, which subsequently collide and form bigger clusters until reaching a critical size to remain in the solution and grow to crystals [31–33]. Besides the spherical cluster formation in the supersaturated solution (homogenous nucleation), formation can also occur on pre-existing substrates, like detrital minerals. The presence of such foreign surfaces can change the kinetic reactions and alter the dynamics of crystal formation [28, 30, 34, 35]. This heterogeneous nucleation is kinetically more favourable [36]. The potential of carbonate crystal nucleation on mineral surfaces can vary due to different interfacial energies among the substrate, solution and precipitates, resulting in different nucleation rates [30]. Natural calcite cementation can take place in the presence of various types of substrates, such as different sediment grains or previously existing calcite cement [37]. In this study, heterogeneous nucleation of calcite in grain packs of different compositions (siliciclastic and calcareous) was targeted with the objective to evaluate the extent of associated nucleation preferences.

## Material and methods

### Sample material

**Fig 1:**
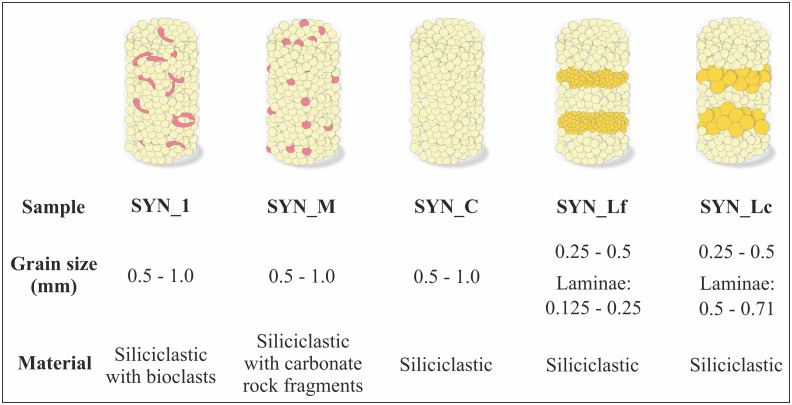
Compositional and textural characteristics of the sample material used for the experiments. Sample sketches not to scale.

Five different natural sand compositions were used in this study with varying amounts of siliciclastic and carbonate grains (S1 Table). The different compositions and grain sizes were chosen to represent a wide range of natural sands and to investigate the effect of grain composition and grain size on the precipitation and distribution of calcite cement. While sample SYN_1 was artificially enriched in bioclasts (biogenic), sands of sample SYN_M are naturally enriched in recycled carbonate rock fragments (inorganic, Fig 1). The other three samples are composed of siliciclastic grains. Sample material has been wet-sieved to the specified grain size ranges and dried in the oven at 50°C. Before using the sand for experiments, the material has been examined qualitatively with a reflected light microscope to check for organic particles. Sand was poured into the individual sample cells (51.5 cm^3^) and carefully shaken for grain rearrangement and denser packing. Sand packs were tightly fixed in the sample cell to ensure that grains do not move when turning the sample during the experiments. The mineralogical composition of the samples was investigated by petrographic analysis. Detailed information on the petrographic analysis is given in the chapter “Analytical methods”.

### Precipitation experiment and flow-through apparatus

**Fig 2:**
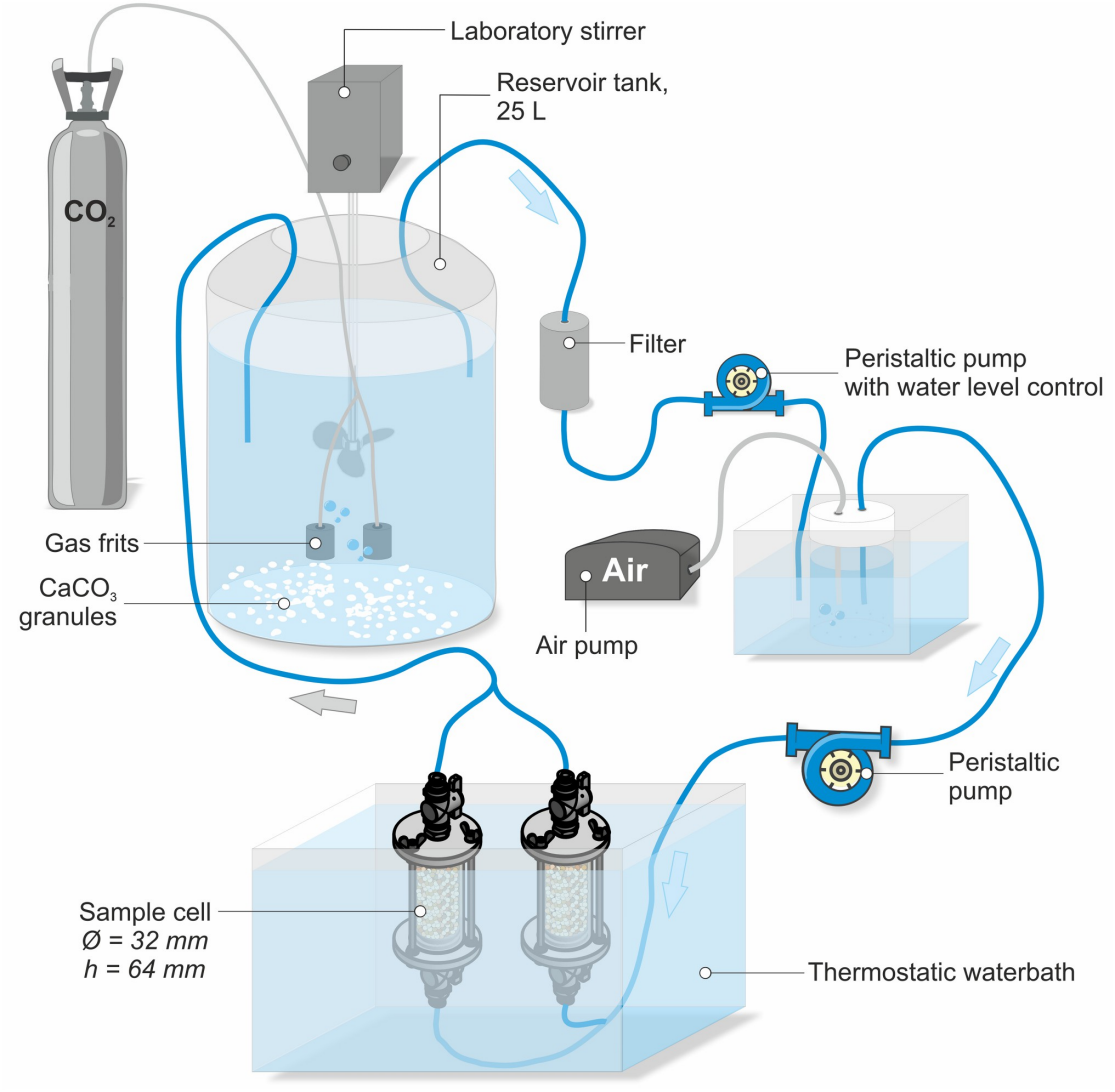
Overview of the experimental setup (schematically simplified). Dissolution of CaCO_3_ granules (98.5% purity) in deionized water by adding CO_2_ in a large reservoir (25 L), followed by filtering through cotton and fiberglass filters and decrease of *p*CO_2_ by adding air to the solution in a smaller vessel. The solution is pumped through the sample cells from bottom to top, and subsequently pumped back to the reservoir.

Precipitation experiments were performed on five samples with different sand composition and grain sizes using an in-house built flow-through apparatus (Fig 2) based on the experimental setup of Cailleau [38] and Molenaar and Venmans [21]. The continuous flow of supersaturated fluid through sand simulates the natural process of calcite precipitation in a sediment body, where sediment grains serve as nucleation sites for the precipitation and growth of calcite cement. All experiments were carried out at room temperature (average of 23°C) and atmospheric pressure (Table 1).

**Table 1:** Summary of the continuous flow experiment. pH and SI values calculated from PHREEQC using experimentally measured parameters before the start of the first experiment.

	infiltration solution
T (°C)	22.8
pH_measured_	6.64
DIC (mmol L^-1^)^a^	25.04
Ca (mmol L^-1^)^b^	8.73
pH_calculated_^c^	6.62
SI^c^	0.54

^a^ Average of 3 measurements.

^b^ Average of 3 ICP-MS measurements.

^c^ Calculated with PHREEQC using the *Thermodem* database at 23°C.

The infiltration solution is obtained in two stages by manipulating the solubility of CaCO_3_ in an open system. The first stage involves saturation of deionized water with respect to CaCO_3_ until a pH (SevenCompact pH meter, Mettler-Toledo) of 6.0–6.2 is obtained:

$$CaCO_{3}+H_{2}O+CO_{2}\to Ca^{2+}+2\;HCO_{3^{-}}.$$ (4)

In the second stage the solution is filtered and transferred into a smaller vessel. In this vessel CaCO_3_ solubility is reduced by decreasing *p*CO_2_ via air prelude until a pH between 6.8 and 7.0 is reached:

$$Ca^{2+}+2\;HCO_{3^{-}}\to CaCO_{3}↓+H_{2}O+CO_{2}↑.$$ (5)

After the required pH is reached, the solution is pumped through the sample cell. The outgoing volumetric fluid flow rate *Q* was quantified by measuring the sampled fluid volume *V*_*fluid*_ and sampling time *t*

$$Q=\frac{V_{fluid}}{t}$$ (6)

The sample cell of the flow-through apparatus has a cylindrical shape with a diameter of 32 mm and a length of 64 mm. The flow rate was set to 17 mL min^-1^ (1 L hr^-1^), which results in a hydraulic retention time (HRT) of 3 min. Every six to twelve hours the sample was taken out of the thermostatic water bath (20°C) and the sample cell was turned manually in order to switch the inlet and outlet connections so, both sides of the sample were equally exposed to the inlet/outlet solution. The duration of the experiment varied between 35 (for a single sample) and 58 days (for two parallel samples) and was stopped when outgoing fluid was blocked, and no flow could be measured. Samples were air-dried for seven days within the sample cell. Afterwards the sample cell was removed carefully and samples were dried in the oven at 40°C for another 24 hours. Samples have been impregnated with blue-dyed epoxy for thin section preparation, after measuring He-porosity (see chapter “Analytical methods”).

### Analytical methods

Initially, fluid samples were taken from different positions of the flow-through apparatus (the reservoir tank where CaCO_3_ is dissolved, and right before the solution enters the sample cell; Fig 2) to calibrate the experiment by measuring concentrations of calcium ions and total inorganic carbon. The concentration of Ca^2+^ ions were analysed using an iCAP Qc inductive coupled plasma mass spectrometer (ICP-MS, Thermo Fisher Scientific Inc., S2 Table*)* fed by a SC-2DXS autosampler (Elemental Scientifics). Samples were measured three times and stabilized by two drops of 65% HNO_3_ Suprapur (relative standard deviations < 1%).

The concentration of dissolved inorganic carbon (DIC) was analysed with an OI Analytical Aurora 1030 W analyser and determined from the signal of the internal nondispersive infrared sensor (NDIR) and a set of calibration standards with known concentrations prepared from analytical (A.C.S.) grade potassium hydrogen phthalate (KHP). Previously, the solution was reacted with 1 mL of 5% phosphoric acid (H_3_PO_4_) at 70°C for two minutes to release the dissolved inorganic carbon (DIC) as CO_2_. This CO_2_ was purged from the solution by helium. The geochemical code PHREEQC [39] with the database “Thermoddem” by Blanc et al. [40] was used to determine the speciation of the carbonate system and to calculate calcite saturation indices (SI).

The calculated values based on the composition of the infiltration solution were used to adjust the experiment, ultimately using the settings listed in the previous chapter.

High-resolution petrographic point-count analysis on polished thin sections of the artificially produced samples were conducted using the Gazzi-Dickinson method [41, 42]. Framework and interstitial components (i.e. cement) were counted until three hundred framework grains were reached with all counts provided as vol%. Laminated samples were counted until five hundred framework grains were reached to obtain a statistically reliable dataset and to cover the thinner layers of the samples. Petrographic data are given as average values if not indicated otherwise. Textural analysis, including grain size, roundness [43], sorting [44] and grain contacts, was performed on 150 grains per thin section. A mechanical stepping stage and the software PETROG (v. 4.7.1.0, Conwy Valley Systems) were used for point-count analysis. The crystal size of calcite cement was determined during microscopic analysis. Grains selected during the point count were classified by type according to Folk [45], Caracciolo et al. [46], Pettijohn et al. [47], Zuffa [42]. Lithic fragments were classified using the criteria of Garzanti and Vezzoli [48] and divided into metamorphic (Lm), sedimentary (Ls) and volcanic (Lv) lithics. The proportion of the free surface that is coated by calcite cement was estimated visually. Grain coat coverage is expressed as the percentage of cement rims on the available grain surface following the approach of Taylor et al. [24] and provided as area%.


$$Grain\;Coat\;Coverage=\frac{\sum Coated}{\sum Coated+\sum Uncoated}\;x100$$ (7)

Scanning electron microscopy (SEM, Hitachi TM4000Plus Tabletop Microscope equipped with an energy dispersive X-ray spectroscope EDX, Oxford Instruments) was used to characterize textural and mineralogical features. Intergranular volume (IGV) was classified according to Paxton et al. [49]. Petrophysical measurements were performed on plugs of three samples (SYN_1, SYN_C, SYN_M), that were oven-dried at 60°C for at least 48 hours before measuring. True (skeletal) density was measured with a Helium pycnometer (AccuPyc II 1340, Micrometrics^®^). Effective porosity was then calculated as the quotient of bulk density (calculated from bulk volume and dry mass) and true density [50].

## Results

### Framework composition and texture

**Fig 3:**
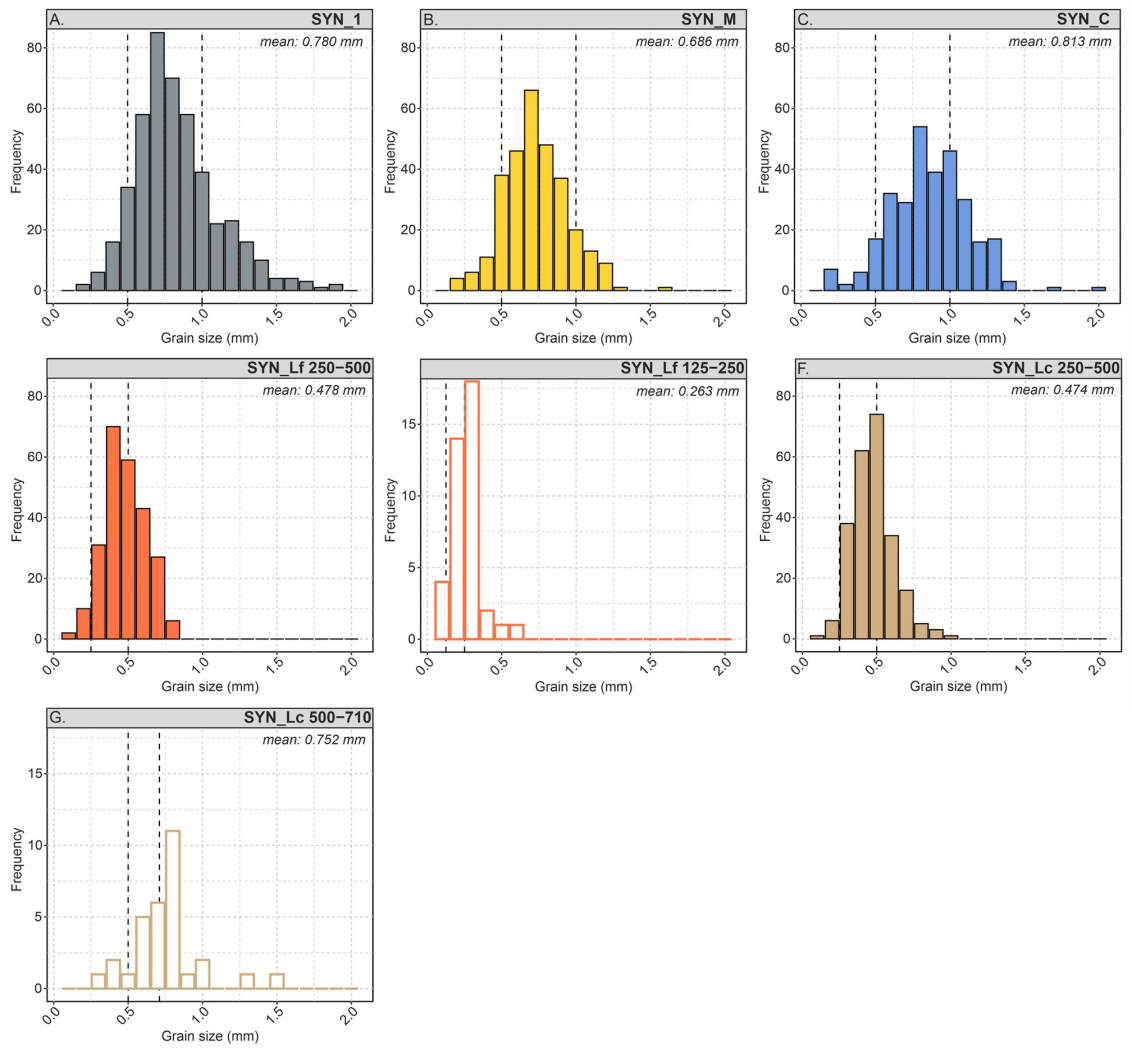
Grain size histograms of the analysed samples collected from petrographic point count analysis. Dashed lines showing the sieve-separated grain size ranges, n = 150 measured grains per sample.

All samples show angular to subrounded grains with moderate to well sorting (Table 2). Sand fractions of the samples SYN_1, SYN_M and SYN_C have been sieve-separated between a grain size of 500–1000 μm (average 0.76 mm, σ = 65.9 μm). Analysed mean grain sizes vary between 0.813 (SYN_C) and 0.686 mm (SYN_M) (Fig 3). Samples SYN_Lf and SYN_Lc are each characterized by two approx. 5 mm thick layers of different grain size and surrounding grains of the same grain size (sieved 250–500 μm, average 0.476 mm). Fine-grained laminae of the sample SYN_Lf show an average grain size of 0.263 mm, while coarse-grained laminae of the sample SYN_Lc show an average grain size of 0.752 mm (Fig 3). Analysed mean grain sizes of the samples SYN_Lf and SYN_Lc are 0.400 and 0.461 mm, respectively.

**Table 2:** Textural data of the analysed samples. n = 150 measured grains per sample.

Sample	SYN_1	SYN_M	SYN_C	SYN_Lf	SYN_Lc
**Grain size (mm)**					
Mean	0.780	0.686	0.813	0.400	0.461
Std.dev	0.255	0.214	0.262	0.158	0.180
**Sorting**					
σ	0.514	0.461	0.495	0.666	0.530
	moderate	well	well	moderate	moderate
**Roundness**	angular-subrounded	angular-subrounded	angular-subrounded	angular-subrounded	angular-subrounded

**Fig 4:**
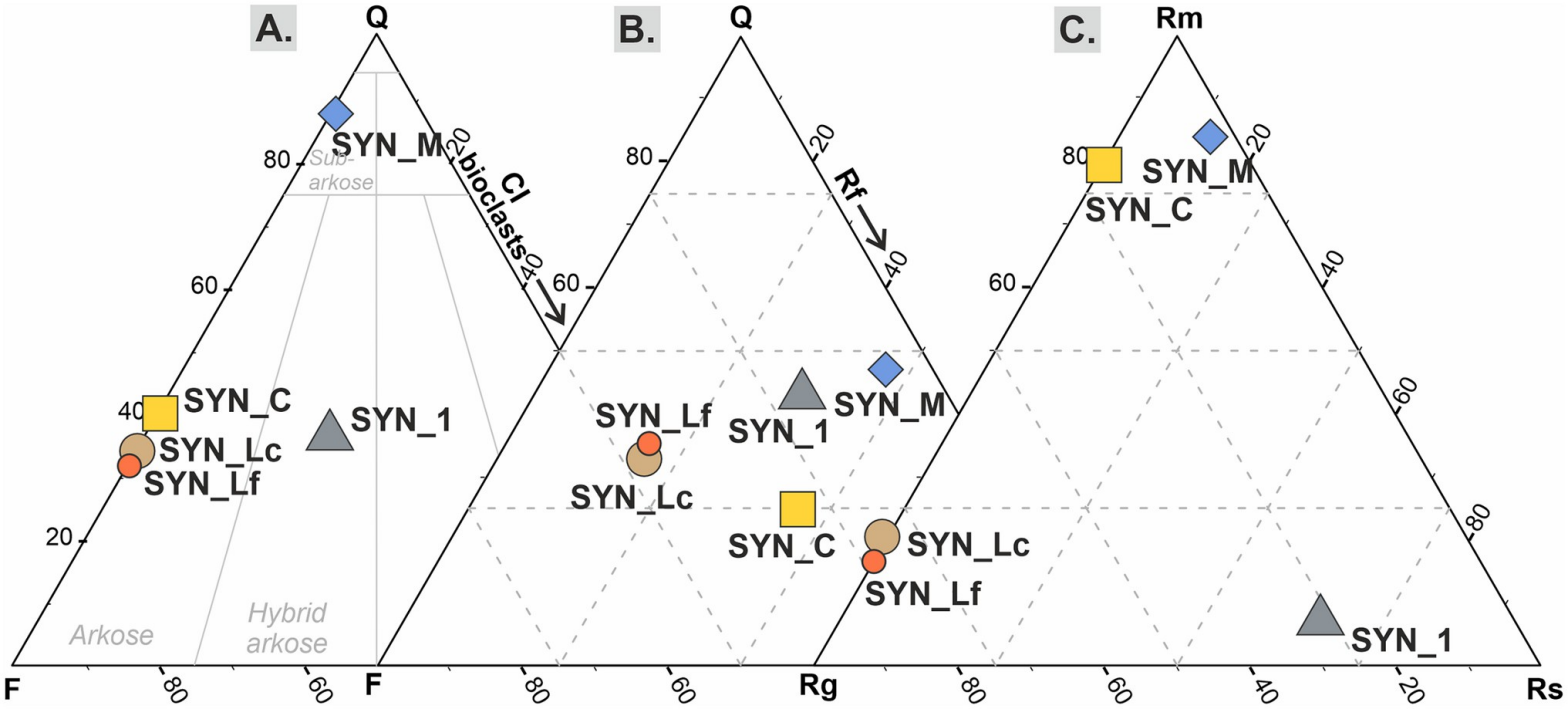
Ternary plots showing the framework composition of the artificially produced samples. A) QFCI with CI (carbonate intrabasinal grains) representing bioclasts [45, 51]; B) QFRf [47]; C) RmRgRs. Q: quartz; F: feldspars; CI: carbonate intrabasinal grains; Rf: rock fragments; Rm: metamorphic rock fragments; Rg: granitic rock fragments; Rs: sedimentary rock fragments. Percentages given in S3 Table.

As one aim of this study is to differentiate the precipitation patterns of calcite in terms of detrital grain composition, the five samples differ in the amount and type of minerals and rock fragments. Samples are dominated by siliciclastic grains (> 41.0 vol% of total components) with only SYN_1 being enriched in bioclasts (biogenic, 10.4 vol%, Fig 4A) and SYN_M enriched in recycled carbonate grains (inorganic, 2.0 vol%) (Table 3). Monocrystalline quartz (18.2 vol% on average) is the most common quartz type in all samples, followed by composite (Qc, max. 6.8 vol%) and polycrystalline (Qp, max. 0.45 vol%) quartz in considerably minor amounts. Potassium feldspar and plagioclase occur in similar abundances (4.3 and 4.0 vol%, respectively) often being altered. Rock fragments are important components as they contribute up to 29.6 vol% of TC (Fig 4B). SYN_1 shows considerable amount of sedimentary rock fragments, including bioclasts (11.1 vol%), and plutonic rock fragments (5.4 vol%). In SYN_Lf and SYN_Lc plutonic rock fragments (8.3 vol%) prevail over metamorphic (1.9 vol%, Fig 4C). Conversely, rock fragments in SYN_M and SYN_C are mostly metamorphic (metapsammite) in composition (24.1 and 22.0 vol%, respectively) with only minor amounts of plutonic fragments (1.0 and 7.4 vol%, respectively).

**Table 3:** Compositional data of the analysed samples. All data in vol% from total counted points (SYN_1 = 582, SYN_M = 498, SYN_C = 500, SYN_Lf = 850, SYN_Lc = 890).

Sample	SYN_1	SYN_M	SYN_C	SYN_Lf	SYN_Lc
**Detrital**					
Qm	14.6	29.1	13.6	17.1	16.7
Qp	0.45	0.0	0.0	0.0	0.0
Qz in PRF	2.3	0.8	2.6	2.9	3.0
Qz in MRF	0.90	23.9	9.0	1.2	1.7
Qz in SRF	0.46	0.0	0.0	0.0	0.0
Qc	6.8	0.0	0.20	0.35	0.56
Kfs	1.6	0.0	1.0	7.5	6.3
alt. Kfs	1.6	1.0	7.6	9.2	7.5
Kfs in PRF	0.91	0.0	2.0	3.1	2.1
Kfs in MRF	0.0	0.0	5.0	0.35	0.34
Pl	2.3	0.0	0.40	0.71	0.0
alt. Pl	4.5	2.8	6.2	12.1	10.9
Pl in PRF	2.3	0.20	2.0	0.94	2.0
Pl in MRF	0.90	0.21	6.6	0.0	0.34
Mica	0.23	0.0	0.40	2.4	2.8
Mica in PRF	0.0	0.0	0.80	0.82	1.7
Mica in MRF	0.23	0.0	1.4	0.0	0.0
Dense Minerals	0.23	0.0	0.0	0.24	0.0
Lm	0.0	0.0	0.60	0.0	0.0
Lv	0.91	0.0	0.0	0.0	0.0
Ls	0.0	0.0	0.40	0.0	0.0
Recycled carbonate grains	0.23	2.0	0.0	0.0	0.0
Bioclasts	10.4	0.40	0.20	0.0	0.0
**Calcite Cement**	**23.2**	**21.3**	**10.4**	**18.9**	**14.5**
Grain-coating	20.5	8.0	7.6	10.9	4.8
Pore-bridging	1.1	6.4	0.40	4.1	4.8
Pore-filling	1.6	6.8	2.4	3.9	4.8
**Porosity**	**25.2**	**18.5**	**29.3**	**22.2**	**25.8**
Intergranular	24.5	18.5	23.9	22.2	25.8
Intragranular	0.68	0.0	5.4	0.0	0.0
He-porosity	38.7	22.9	33.2	-	-
**IGV**	**47.7**	**39.8**	**34.3**	**41.1**	**40.3**

Qm: monocrystalline quartz, Qp: polycrystalline quartz, Qz: quartz, Qc: composite quartz, Kfs: K-feldspar, alt. Kfs: altered K-feldspar, Pl: plagioclase, alt. Pl: altered plagioclase, PRF: plutonic rock fragments, MRF: metamorphic rock fragments, SRF: sedimentary rock fragments, Lm: metamorphic lithics, Lv: volcanic lithics, Ls: sedimentary lithics, IGV: intergranular volume.

### Distribution, occurrence and morphology of calcite cement

**Fig 5:**
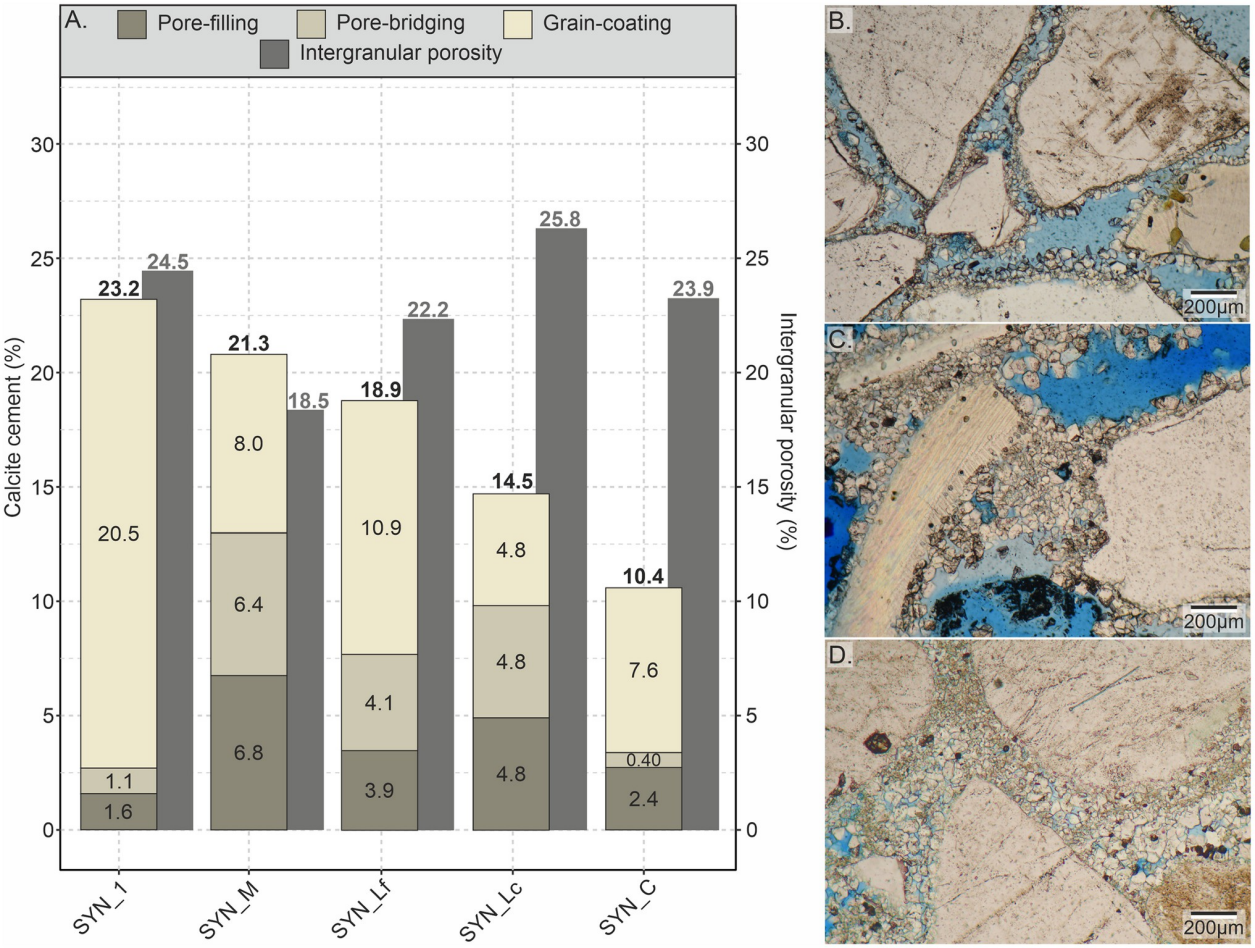
Calcite cement types and distribution. A) Volume proportions of calcite cement types (total vol% of calcite cements as bold number) and intergranular porosity in the produced sandstones with microphotographs in plane-polarised light of B) grain-coating, C) pore-bridging and D) pore-filling calcite cement.

**Fig 6:**
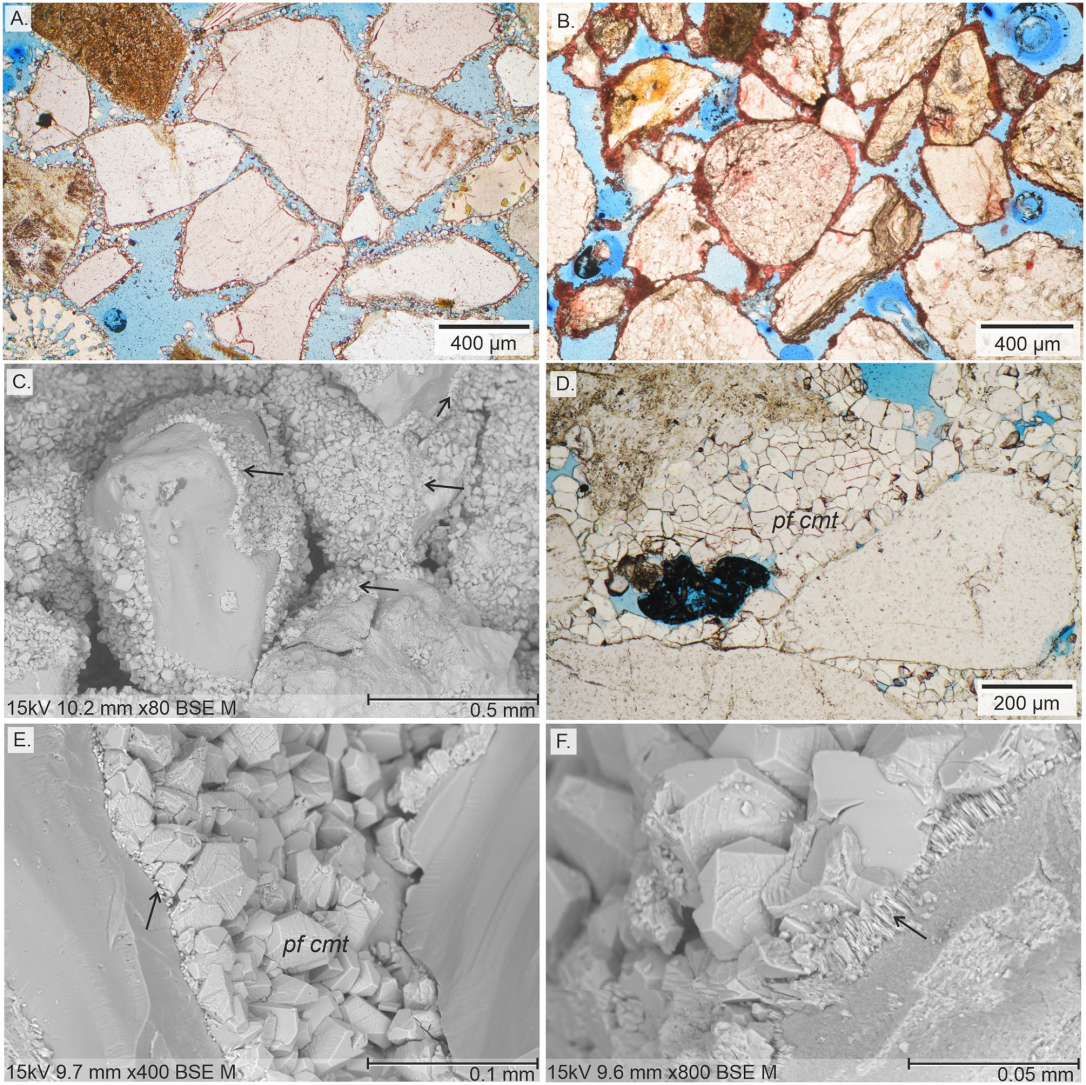
Optical and scanning microscope images of precipitated calcite cements. A) Microphotographs of sample SYN_1 with fine, grain-coating calcite crystals on siliciclastic and bioclastic grains, similar to B) Grain-coating calcite cement of a natural beach sample (BEL, see [52]); C) SEM image of siliciclastic grains covered by grain-coating calcite cement (arrows, SYN_M); D) Relatively coarse-crystalline pore-filling calcite cement (pf cmt) in sample SYN_M; E) Well developed pore-filling calcite cement (pf cmt) on siliciclastic grains (note that the grain surface of the left grain was cut during thin section preparation) with small crystals along the grain surface (arrow) which increase in size towards the open pore space (SYN_1); F) Calcite with well-developed crystal surfaces and very fine, fibrous crystals (arrow) directly on the grain surface (SYN_1).

A continuous precipitation of calcite cement was observed throughout each flow-through experiment. Microscopic examination of the produced synthetic samples shows the precipitation of granular calcite with an average crystal size of about 30 μm. Along the full size of the sample plug, the precipitation of calcite cement is not uniform. The highest cement abundance is observed in areas located close to the inlets of the cell. The artificially cemented sand samples contain between 10.4 and 23.2 vol% of total calcite cement (Fig 5A). The following textures of authigenic calcite have been observed from thin section analysis: 1) grain-coating, 2) pore-bridging, and 3) pore-filling calcite (Fig 5B–5D). The observed textures are similar to those of natural samples, for example from beach environments of Calabria (compare Fig 6A and 6B, Janssen et al. [52]). The occurrence of cement textures varies significantly among the samples. While grain-coating cement is clearly dominant in samples SYN_1 (88.4 vol% of total cement, Figs 5A, 6A and 6C) and SYN_C (73.1 vol% of total cement) followed by pore-filling cement (6.9 and 23.1 vol%, respectively), the other samples show a more balanced occurrence of cement types, particularly SYN_M and SYN_Lc with almost equal percentages of grain-coating, pore-bridging and pore-filling cements (Fig 5A). Individual crystals consist of euhedral calcite with a size of 4 to 100 μm. Average crystal size is coarsest in the samples SYN_C (44.6 μm, σ = 13.1 μm) and SYN_M (42.5 μm, σ = 12.4 μm, Fig 6D). Other samples have a similar average crystal size of about 20 μm. Often, crystal size is finer on grain surface and increases in size towards the open pore space (Fig 6E). In rare cases, crystals directly attached to grain surfaces show a very fine, fibrous texture (Fig 6F). Grain-rims are composed of single or several layers of calcite crystals expanding into the pore space with a thickness between 10 to 200 μm (Fig 5B).

### Composition and texture of substrate grains

Petrographic analysis show variations in the total volume of calcite cement depending on detrital grain composition and average grain size of the substrate material.

**Fig 7:**
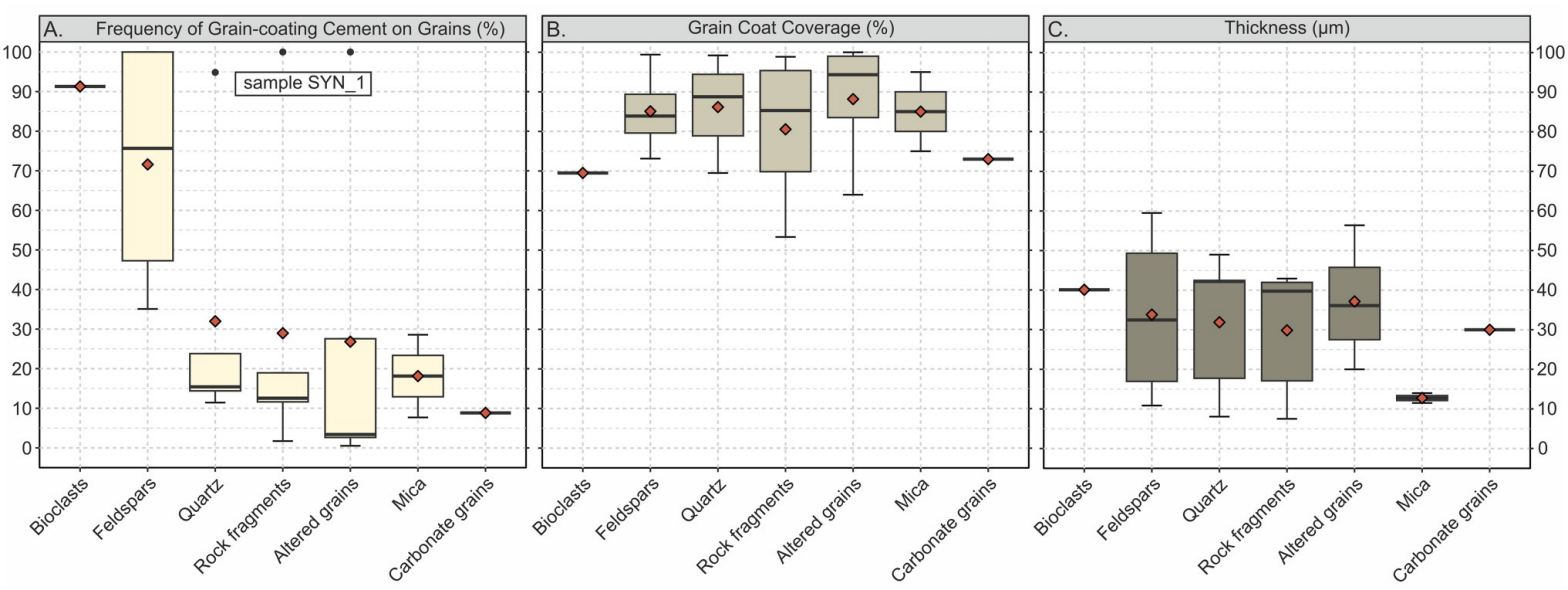
Boxplots showing data of calcite grain-coating cement. A) frequency of calcite grain-coating on detrital grains (ratio of covered grains by total grain number); B) grain coat coverage by calcite cement; C) measured thicknesses of grain coating coverages on grains by grain types. Red diamonds indicate the mean value. Data derived from all experiments–number of covered grains: bioclast = 42, feldspars = 84, quartz = 214, rock fragments = 119, altered grains = 36, micas = 8, carbonate grains = 3. See S4 Table.

To evaluate the effect of detrital composition on the heterogeneous nucleation of calcite crystals on different grain surfaces, grain-coating cements were analysed in terms of how often specific grain types (e.g. quartz, feldspars, bioclasts etc.) are covered by calcite cement (frequency of grain-coating cement on grains), the proportion of detrital grain surface that is covert by calcite cement (grain coat coverage, see Analytical methods) and the thicknesses of grain-coating cements. Naturally, the relationship of total grain-coating cement and the frequency of grain-coating cement on the detrital grains is linear. Meaning, the more grain-coating cement precipitated in a sample, the more detrital grains are covered by cement. However, there is no relationship between the total abundance of particular detrital grain type compositions (e.g. quartz, feldspar, bioclasts etc.) in the individual samples and the frequency of grain-coating calcite cement on these grains. Which means that although a specific grain type is particularly abundant in a sample, this kind of grain type is not necessarily most frequently coated by calcite cement, in turn implying independence between those two variables. For example, feldspar (Ksp+Plag) grains show a low to intermediate abundance (0–8.2 vol% of TC) in all of the samples. The proportion of feldspars showing grain-coating calcite cement on their surfaces, however, is higher than for other grain types which are more abundant (median of 75.7%, Fig 7A). Although, the range of grain-coating cement frequencies on feldspars is rather large in all five samples. Conversely, quartz is one of the most abundant grains but shows a median frequency of grain-coating cement of 15.4%.

**Fig 8:**
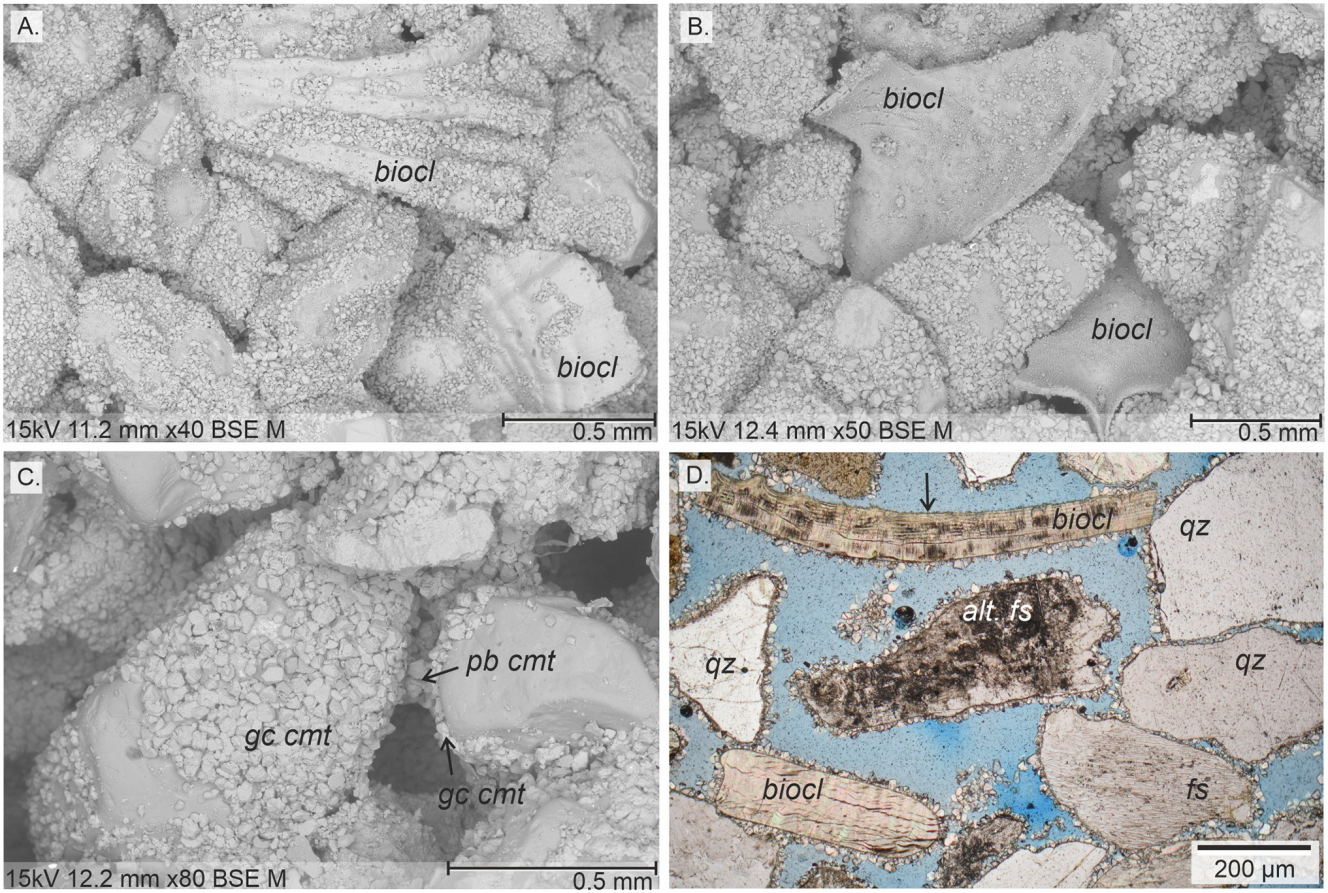
Optical and SEM images of calcite cements on different grain types. A) and B) Though almost all bioclast (biocl) counted show grain-coating cement, they tend to have less continuous grain coat coverage of calcite and smaller crystal sizes compared to other grain types. Images from experiment SYN_1; C) Siliciclastic grains covered by coarse calcite cement (gc cmt, averaging crystal size of 50 μm, SYN_M) with pore-bridging cement between two grains (pb cmt); D) Various siliciclastic grains (quartz–qz; feldspar–fs; altered feldspar–alt. fs) with complete grain coating coverage. The lower bioclast (biocl) also shows a high coverage, while the upper bioclast is only partly covered (arrow indicating non-coverage).

All grain types show grain-coating calcite with bioclasts being coated most frequently (91.3%), followed by feldspars (75.7%, Fig 7A). Other siliciclastic grains are coated less frequently from a median of 15.4% for quartz to 3.4% for altered grains (mostly feldspars). Sample SYN_1 shows exceptionally high values of calcite grain-coating cement for all contained grain types (97.2% on average) being the sample with the highest amount of total calcite cement (Fig 5A). Recycled carbonate grains are by far the least frequently coated grains with only 8.8%. However, both, bioclasts and reworked carbonate grains, show the least coverage with similar values of 69.5 and 73.3%, respectively, (Figs 7B and 8A, 8B and 8D), while siliciclastic grains have grain coat coverages ranging from 94.3 to 83.9% (Figs 7B, 8C and 8D). Particularly altered grains, mostly consisting of plagioclase, show high coverage (94.3%) of calcite cement.

As shown in Fig 7C, grain-coating cements on bioclasts reach an average thickness of 40.1 μm, followed by altered grains (mostly feldspars, 37.2 μm) and feldspars (33.8 μm). Recycled carbonate grains are covered by calcite with an average thickness of 30 μm, joining the rank of the siliciclastic grains, although, less frequently covered (Fig 7A). Calcite cement on micas is by far the thinnest reaching an average thickness of only 12.8 μm. However, the variance of cement thicknesses on the different siliciclastic grain types is rather high with variations of up to 20 μm for feldspar and slightly less for quartz, rock fragments and altered grains (Fig 7C).

**Fig 9:**
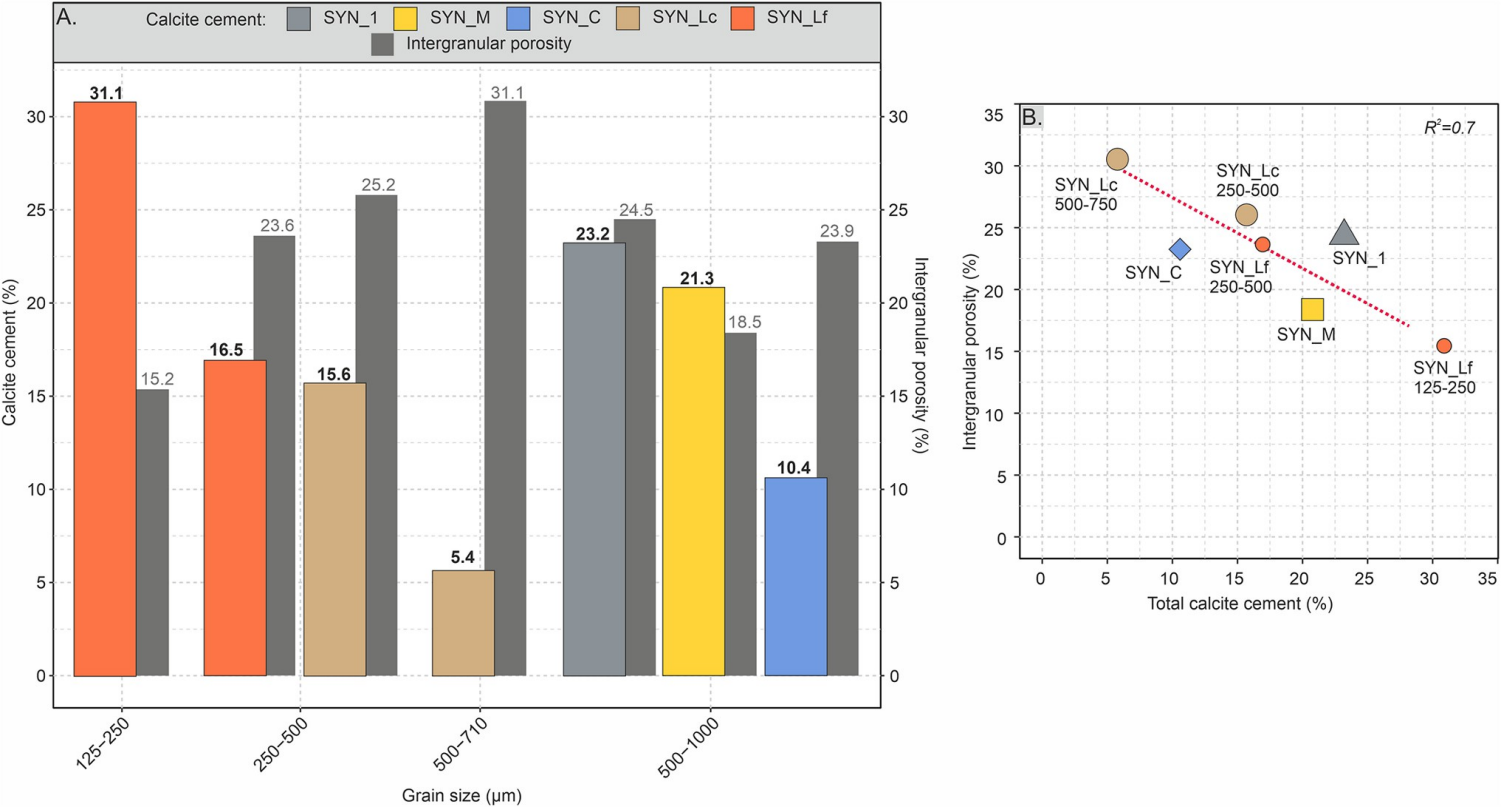
Calcite cement and intergranular porosity volumes in sand packs of different grain size. A) Distribution of calcite cement and intergranular porosity percentages in different detrital grain size fractions; B) Relationship of total calcite cement contents and intergranular porosities showing a negative correlation of R^2^ = 0.7. Data of the laminated samples SYN_Lc and SYN_Lf are given for the different grain size laminae.

Laminated samples with same detrital composition but differing mean grain sizes were used to test whether the precipitation of total calcite cement is influenced by grain size (SYN_Lf, SYN_Lc). Calcite is most abundant (28.2%) in the finest layers, which have a grain size range of 125–250 μm and least abundant (6.3%) in the coarsest layers of 500–710 μm grain size (Figs 8D and 9A). The intermediate grains size range of 250–500 μm was tested in both samples and shows consistent results with a calcite cement abundance of 16.3 (± 0.6) % on average.

### Porosity evolution

Results of 2D microscopic porosity measurements for all samples show total porosity values between 18.5 and 29.3% (Table 3). Highest porosities are in the sample SYN_C, however, about 5.4% can be ascribed to intragranular porosity of altered grains. Other samples only have minor intragranular porosities (< 1%). Intergranular porosity reduction is attributed to the precipitation of calcite (Fig 9B). While samples SYN_M, SYN_Lf and SYN_Lc show a very good negative correlation of cement content and porosity (R^2^ = 0.9), samples SYN_1 and SYN_C do not align with this trend and show respectively higher and lower porosity values than expected. The samples show no petrographic evidence consistent with secondary porosity formation.

### Saturation index

**Fig 10:**
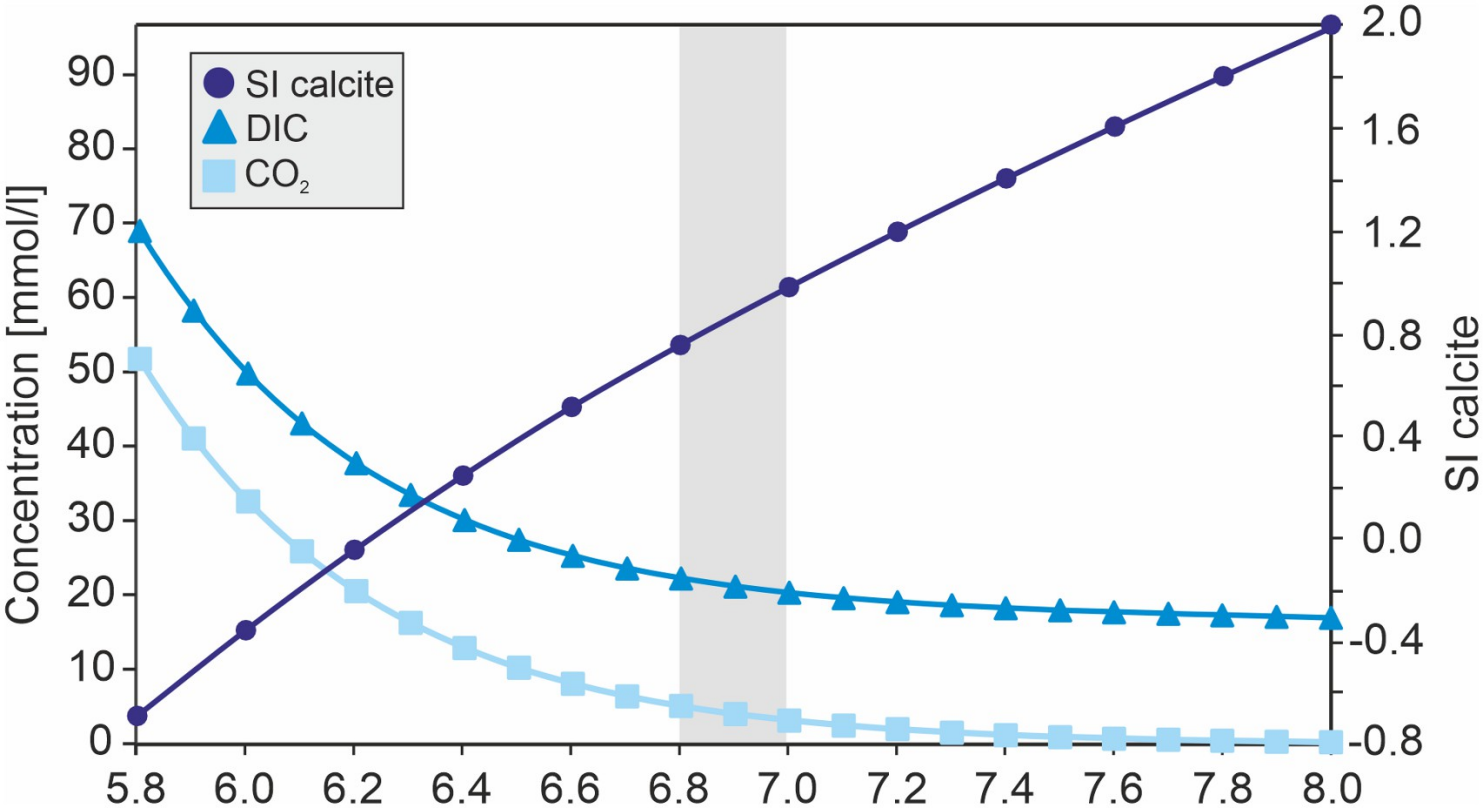
PHREEQC model of saturation index and concentration of dissolved inorganic carbon and CO_2_. Saturation index calculations of calcite and concentration of total dissolved carbon (DIC) and dissolved CO_2_ as a function of pH of the infiltration solution. pH values of 6.8–7.0 for the solution entering the sample cell show a SI between ~0.7–0.9 (highlighted in light grey shading) indicating a clear supersaturation of the solution. Solutions of the reservoir (pH of 6.0–6.2), where CaCO_3_ has been dissolved show SI values below or close to equilibrium.

Calcium and DIC concentration of the infiltration solution was measured in advance to calibrate and improve the experiment (Table 1). On the basis of the measured calcium and DIC concentrations, SI calculations are simulated by forcing the solution to pH values between 5.8 and 8.0 and for considerations on the dissolved carbon species. SI calculations show that the infiltration solution reaches equilibrium at a pH close to 6.2. The solution is supersaturated with respect to calcite right before it enters the sample cell (pH 6.8–7.0) with SI values between 0.76 and 0.98 (Fig 10). In Fig 10 the concentration of the dissolved carbon species as a function of pH is shown, indicating a steep concentration reduction until a pH of about 6.4 is reached. This development corresponds to the precipitation of calcite within the solution.

## Discussion

### Crystal distribution and fluid flow paths

In all experiments, precipitation and crystal growth of CaCO_3_ was not homogenous. Near the plug inlets more crystals precipitated than in the centre part. This finding is consistent with results obtained from other studies of mineral precipitation in porous media [53–58]. Such a heterogeneous crystallisation pattern might be attributed to a gradient of supersaturation that developed inside the porous media, with decreasing supersaturation from the inlet points towards the centre of the sample cell. The development of non-uniform cementation might have been further promoted by the preferential nucleation of calcite on initial crystals due to lower interfacial energies [59]. Furthermore, fluid flow velocity can influence surface charge, which can have a significant impact on nucleation on particle surfaces and overall reaction rates [60, 61]. As precipitation of calcite cement proceeds, the available pore space for fluid flow declines. Therefore, fluid velocities and residence times within the sample will change over time, which might have influenced a heterogeneous distribution of calcite cement in the samples.

### Crystal morphology and growth

The produced calcite cement consists of fine, euhedral crystals with a size between 4 to 100 μm. Crystals of more than 100 μm or different textures, like syntaxial overgrowths or poikilotopic cements, were not observed. CaCO_3_ crystal morphology and growth in natural environments depends on the degree of saturation, precipitation rate [62–64], potential microbial mechanisms [14, 65–69] and the concentration of certain ions in the solution, mainly Mg^2+^ [28, 64, 70–72]. The latter consideration can be neglected for the presented experiments as ionic impurities of the solution have been avoided. Bosak and Newman [69] investigated the effects of supersaturation on calcite morphology, stating that at high saturation indices the total number of crystals increases and crystal sizes decreases due to an increase in the number of crystal nuclei. This observation coincides with the classical nucleation theory assuming a constant and small critical supersaturation for nucleation in nature [64, 73]. Carbonate nucleation in natural systems presumably occurs close to equilibrium in most cases [64, 74, 75]. The formation of poikilotopic cements most likely occurs in pore solutions of very low concentrations and at relatively slow rates [76–78]. The calcite crystal morphology in the experiments, by contrast, reflects relatively high supersaturation and short experimental time at low temperature and pressure. Higher temperature would probably result in larger crystals as growth rates of already existing crystal nuclei would be faster than the nucleation rate of new crystal nuclei [79].

### The role of grain and pore size

**Fig 11:**
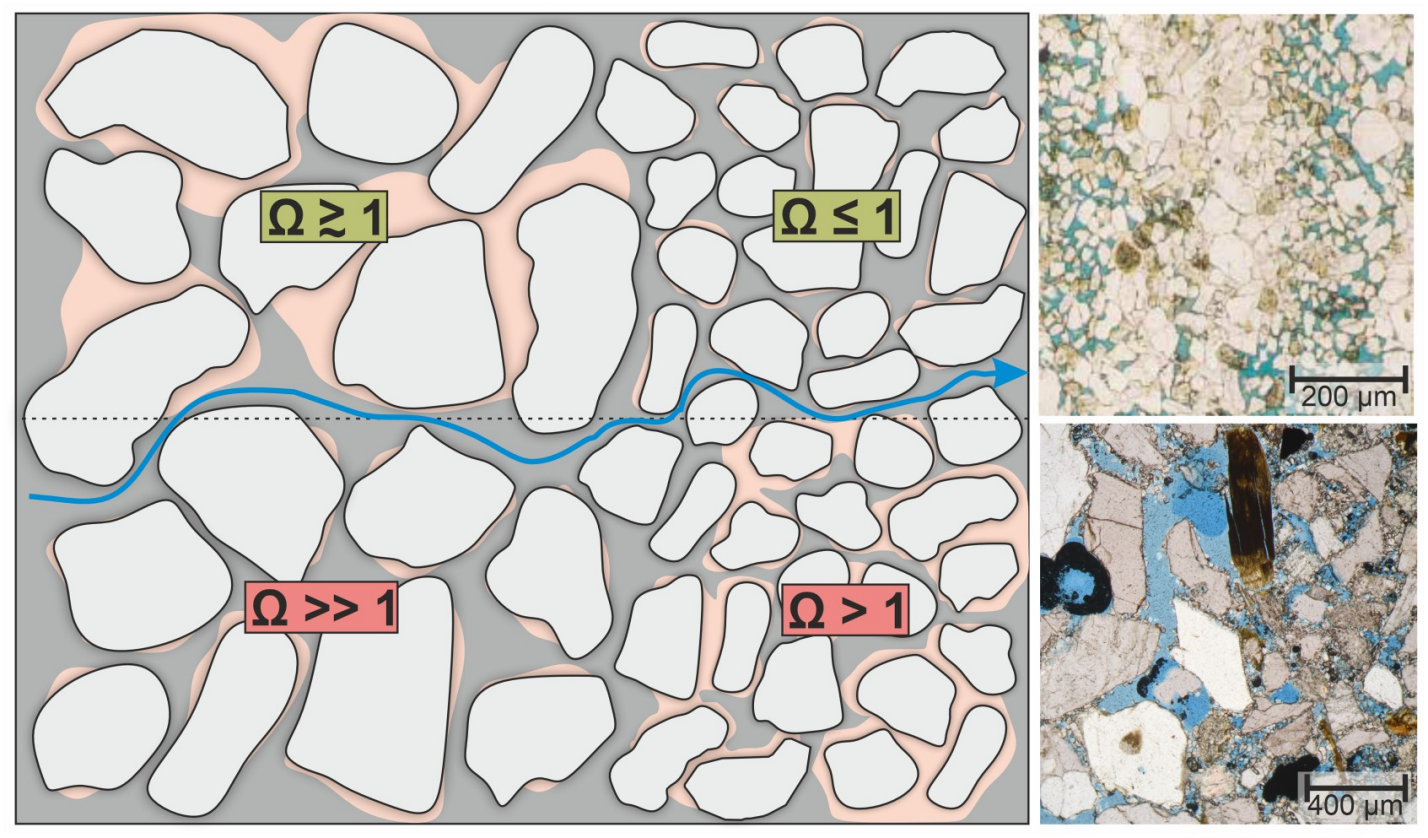
Illustration of pore-size controlled solubility (PCS). PCS in natural systems close to equilibrium (top, green) and experimental conditions far from equilibrium (bottom, red) with thin section images of cements in sandstones for comparism (top image: halite-cemented laminated sandstone from Putnis and Mauthe [81]; bottom image: experiment SYN_Lf from this study). The effect of PCS reduces the supersaturation from larger pores in coarse-grained laminae to smaller pores in fine-grained laminae. In nature, the increase in solubility in smaller pores creates saturation or undersaturation resulting in no or rare precipitation of cement. In the laboratory, fluids are far from equilibrium meaning PCS cannot explain the observed cement distribution. Sketch: Ω: saturation state, grey colour–pore space, pink–authigenic cement, blue arrow–fluid flow; microphotographs: blue colour–pore space (blue-dyed epoxy).

Results of layered samples show greater cement volumes in fine-grained layers with less cement in coarser laminae. This observation is in contrast with preferential cementation of calcite [6, 80] and other mineral phases, like halite or quartz [81, 82], in larger pores of natural systems. In many cases this precipitation pattern is explained by “pore-size-controlled solubility” (PCS), which occurs as a result of surface tension associated with crystals growing in confined pores [81–83]. With decreasing pore size, interfacial energy effects and increasing crystallisation pressure cause changes in solubility and more extensive dissolution of clusters or crystals. Therefore, crystal size is limited by the dimension of the pores with the effective solubility being inversely proportional to pore size [83–85]. Thus, saturation indices that promote precipitation in large pores, will not lead to cluster formation in small pores as the solubility will increase (Fig 11 top). However, PCS has mostly been investigated in small pores (< 40 μm) or micron- to nanometre scale pores and the impact on overall precipitation rates is most distinct in systems close to equilibrium with extremely low supersaturation [82, 86]. Therefore, even minor variations in supersaturation due to changing pore size domains can result in a change from precipitation to non-precipitation and vice versa.

Cementation patterns of the synthetic samples SYN_Lf and SYN_Lc are in contrast with the above-mentioned observations. One reason for this could be the high degree of supersaturation, which is several times higher than the equilibrium. Assuming that PCS influences the saturation state of the pore fluid and precipitation occurs due to small changes ‘close to equilibrium’, systems with highly supersaturated fluids will not be affected as even a change in pore size will not change the supersaturated character of the fluid (Fig 11 bottom). Therefore, it is most likely that pore-wall surface area is the dominant control on cement volume as expected from standard kinetic theory, where the ratio of surface area to volume increases with decreasing pore size, meaning that heterogeneous nucleation will increase as pores become smaller [27, 87–89].

**Fig 12:**
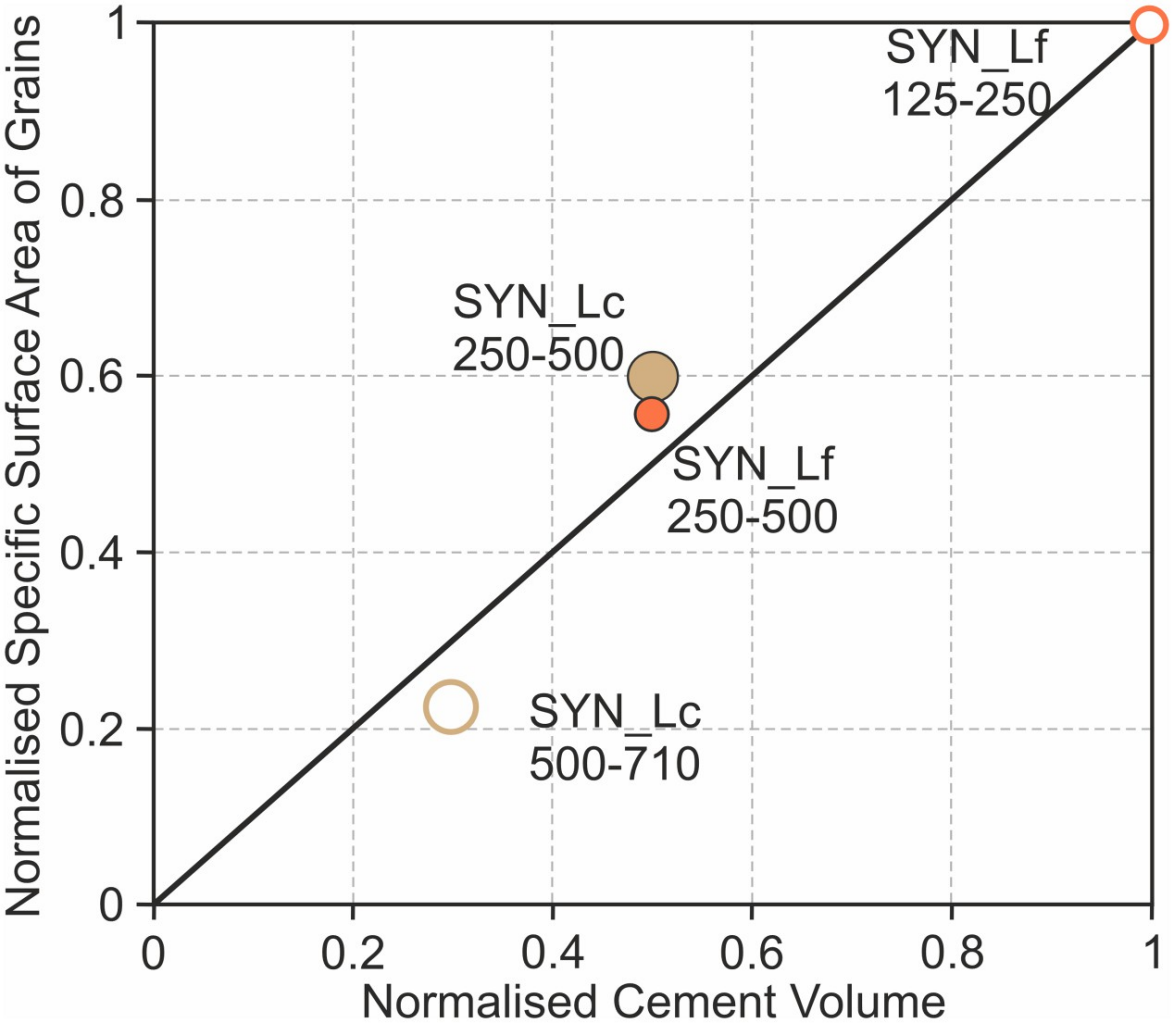
Correlation of cement volume and specific surface area of grains. Plot showing the normalised cement volume (1 = 28.2 vol% calcite cement of the fine layers of sample SYN_Lf) against the specific surface area of grains(area/volume) based on the mid-point of the grain size ranges for the different layers with values normalised to the fine layers of SYN_Lf (1 = 32 mm^2^/mm^3^). All points plot reasonably close to the 1:1 reference line, illustrating a good correlation between observed cement volume and what can be expected for the available the surface area in the specific layers.

This can also be confirmed by the comparison of the cement volume with calculations of the specific surface area of grains for the laminae in the samples SYN_Lf and SYN_Lc. Fig 12 shows cement volume normalised to the cement content of the fine laminae of sample SYN_Lf (1 = 28.2 vol% calcite cement) against the calculated specific surface area of spheres (area/volume) based on the median value of the grain size ranges of the different layers. Values for the specific surface area are also normalised to the values of the fine laminae of sample SYN_Lf (1 = 32 mm^2^/mm^3^). All points representing the different layers of the two samples SYN_Lf and SYN_Lc are plotting close to the 1:1 reference line showing that the cement volumes variations observed in the samples are close to what would be expected for the surface area variation among the layers. While the amount of cement and the calculated surface area of the medium-grained laminae match well for both samples, coarse laminae seem to have less cement than expected due to the surface area effect. This might indicate additional grain-size related controls on cementation or reflect material loss during sample extraction and transportation due to the fragile nature of the coarsest layers.

Other samples have been prepared with the same grain size range (500–1000 μm), however, do show slightly different mean grain sizes. The observation that the sample with the finest average grain size (SYN_M, 0.686 mm) does have about twice as much cement as the coarsest sample (SYN_C, 0.813 mm) does support the result of the laminated samples and emphases the importance of available surface area for nucleation and cementation. On the other hand, sample SYN_1, which shows an average grain size of 0.780 mm, does not line up to this trend. This might be related to the high abundance of bioclast with variable grain shapes (e.g. elongated shell fragments), which cannot be approached in the same way as the reasonable assumption of spherical grains in the other experiments. Another aspect that could influence precipitation distribution in sand packs of different grain size might be turbulence differences of the fluid within the sample. In the case of laminated samples, the transition of different grain sizes within a sample could influence the turbulence of the pore-fluid leading to enhanced precipitation in areas with higher turbulence [90, 91]. Fluid flow simulations of the experiments could help investigate this aspect.

Sample SYN_1 is characterised by a strong dominance of grain-coating cement. One reason could be a higher IGV, which displays the distance between grains within the sample. As no compaction was induced to any of the samples, the IGV indicates the initial porosity. Therefore, the initial porosity of the samples SYN_1 must have been higher than in the other experiments, impeding the formation of pore-filling calcite as precipitation took place on the grain surfaces rather than growing outwards to the greater open pore space. This could be associated with less dense packing of the sand due to bioclasts and slightly higher modal grain size values for this type of grains (1.2 mm). However, compositional effects due to the presence of carbonatic grains in the samples SYN_1 and SYN_M have to be considered.

### Influence of substrate composition

The probability of nucleation and crystal growth is strongly affected by the value of the critical nucleus size, which is a function of the interfacial energy. The smaller the interfacial energy, the more likely heterogeneous nucleation becomes [92]. The presence of particles, like grains, can lower the interfacial energy resulting in an increase in nucleation rate, following the principles of the classical nucleation theory [36, 92]. In natural systems of low supersaturation or diffusion-controlled processes, nucleation of carbonate cement is often associated with particles showing a strong interaction and good structural match between crystalline phase and substrate. This applies, for instance, to detrital carbonate grains, as the nucleation barrier is high and needs to be reduced to accelerate the kinetics [36].

Our experiments with detrital carbonate material (SYN_1 and SYN_M) have the highest volumes of calcite cement. Furthermore, when bioclasts are used, these grains are most frequently coated by calcite (Fig 7A), but with less grain coat coverage compared to siliciclastic grains (Fig 7B). In the experiment with recycled carbonate grains, calcite did not selectively precipitate on carbonate surfaces. These observations contradict each other and cannot be simply explained by the classical nucleation theory. Several authors conducted experiments to investigate the influence of substrate mineralogy on the precipitation of CaCO_3_ [20, 58, 59, 93, 94]. Most studies found that calcite preferentially nucleates on calcite seeds compared to aragonite or silicate material with only single individual crystals evolving on non-calcite surfaces. The bioclast components in our experiments consist of both calcitic and aragonitic compositions, which have not been differentiated during point counting. Therefore, the less continuous grain coat coverage and sometimes smaller crystal size of calcite cement (Fig 8B) might be attributed to aragonite components. Another mechanism that can influence nucleation is the alteration in surface and solution chemistry due to the presence of organic matter on bioclasts, which can already be effective at relatively low concentrations [95, 96]. Multiple microorganisms are known to induce carbonate precipitation through different physiological activities or by serving as crystal nucleus. They can take up and secrete kinetic inhibitors, bind calcium and magnesium ions on their negatively charged surfaces or influence the SI of calcium carbonates [97–100]. Such mechanisms are particularly important in systems not highly supersaturated with respect to calcium carbonate, such as modern seawater, marine sediments or soils [65, 101, 102]. Although, natural sample material has been carefully cleaned before the experiments, the influence of organic matter should be considered, particularly in sample SYN_1. Minor amounts of organic matter could have been trapped in micropores of the used marine bioclast. This could have influence on the precipitation mechanisms of calcite, which might be reflected by the strong dominance of grain-coating cement texture (Fig 5). The contamination of organic matter on marine bioclast might also explain why sample SYN_1 does not line up in the trend of calcite nucleation in relation to grain size. The influence of organic matter could distort the importance of available surface area on nucleation. Nevertheless, we believe a systematic error in maintaining sterile conditions would have effected all samples or multiple grain types, however, the abovementioned features have only been observed for bioclasts in samples SYN_1. Experiments with calcite-cemented sand, similar to our recycled carbonate grains, show that surfaces did not induce precipitation [58].

Other experiments show that although initial nucleation is faster in the presence of calcite grains, precipitation rates become independent of the substrate over time (after 10 days) with silicate surfaces being extensively covered [94]. As our experimental time exceeds this short time span considerably, the effect of substrate mineralogy on calcite nucleation might be lost. Furthermore, the nucleation barrier becomes lower at high supersaturation and surfaces with a weak interaction and poor structural match are kinetically as favourable as surfaces with good structural match [36].

In addition, multiple other factors can affect heterogeneous nucleation of CaCO_3_ including surface chemistry, roughness, hydrophilicity and surface charge of the substrate [59, 103, 104], which are difficult to isolate and could not been further investigated in this study.

### Simulating nature in the laboratory

The experiments presented in this study were conducted under low temperature and pressure conditions to reflect early (near surface) calcite cementation in samples which have not experienced burial. When compared to natural samples which are characterised by early carbonate cementation, cement’s morphology, texture and small-scale distribution are similar to the synthetically produced cements (Fig 6A and 6B). In nature, possible sources of calcite cement are described as either internal, e.g. from the dissolution of metastable grains, or externally derived from advection of different types of interstitial water, such as meteoric groundwater, sea water or brines [3, 105–108]. Furthermore, microbial-related processes and the degradation of organic matter can facilitate carbonate crystal formation by metabolic activities or EPS-mediated (extracellular organic matter) mineralization [109]. The flow-through experiment is designed to resemble an external source of calcite by the circulation of a supersaturated fluid. During early diagenesis at shallow burial depth, sediments have a high potential to react with depositional pore waters, derived from rain fall, sea water or groundwater [110]. The precipitation of authigenic minerals is thereby limited by the ion concentration of the solution and its fluid flow. In case of near-surface flow of meteoric or marine water, the pore water is constantly renewed and relatively high flow rates can be expected. High porosity and permeability of sand near the depositional surface also allows for large fluid fluxes. However, if the fluid flux is low at shallow depth, pore waters in clastic sediments can be assumed to be close to equilibrium with carbonate minerals due to a high kinetic rate for calcite precipitation [108]. If the pore water is stationary or rock buffered, precipitation requires the dissolution of other minerals, as only small amounts can precipitate before the pore water attains equilibrium [111]. Therefore, a fluid supersaturated with calcite and a sufficient fluid flow are necessary to produce adequate amounts of calcite cement. Such conditions can typically be found in shallow marine environment creating beachrocks [112]. Additionally, carbonate cement may precipitate in response to the mixing of fluid that has percolated through soils with high *p*CO_2_ conditions with alkaline groundwaters that have experienced silicate hydrolysis [113]. Finally, carbonate precipitation due to highly concentrated pore water also may occur in dry environments due to high evaporation rates [107, 114] or distal-to-source environments due to progressive concentration of Ca and Mg [105, 115]. Although, precipitation in such environments can be very rapid with time scales in the order of years to decades [112, 116, 117], laboratory experiments need to find ways to generate results that approximate nature at much shorter timescales. The continuous flow method applied in this study allows to precipitate considerable amounts of calcite cement similar to natural systems in days by circulating a strongly supersaturated solution. Consequently, the experimental conditions cannot reflect environments with low or stagnant fluid flow, where pore fluid are close to equilibrium in respect to carbonate minerals, or general systems close to saturation, as is often the case in geological systems, both at shallow and burial depths [76, 108, 118]. Furthermore, the experiments of this study represent a simplification of reactions with constant temperature, pressure and supersaturation values and no influence of other ions or microbes.

## Conclusion

This study investigates a set of experiments to obtain detailed analysis of calcite precipitation in sands with various compositions and textures. These experiments successfully reproduce early near-surface calcite cements with morphologies that are comparable to naturally occurring cements. Petrographic analysis provides information about the distribution and occurrence of calcite cements. Despite the same chemical and flow conditions, the distribution of new calcite crystals varies depending on the substrate material. Although the influence of substrate composition is difficult to isolate, results demonstrate that the spatial distribution of different components should be taken into account for estimations of precipitation. Our experiments show that both, siliciclastic and carbonatic grains, serve as nucleation sites for calcite cement. While particularly bioclast are most frequently coated by calcite, the grain coat coverage is less good as for siliciclastic grains. Furthermore, detrital grain size strongly influences the pore geometry and leads to different precipitation patterns, indicating the importance in the development of heterogeneous cementation during the very early (near surface) stages of diagenesis.

The presented experiments cannot fully reproduce natural diagenetic conditions due to short experimental time compared to geological time scales and the necessary simplifications of the systems. Nevertheless, this study provides relevant insights into the precipitation processes of calcite in complex porous materials that reflect analogues of natural sedimentary environments with heterogeneous shapes and pore connectivity influencing flow and transport properties, and thereby precipitation. Additional complexity is gained by the heterogeneous distribution of reacting mineral phases. Furthermore, our investigations highlight the importance of the supersaturation state of the infiltration solution, which can be particularly relevant for Aquifer Thermal Energy Storage (ATES), hydrothermal energy production or carbon capture and storage (CCS) systems where brine chemistry has to be adjusted accurately.

Further investigations on systems with different composition and texture, and higher porosity decrease due to calcite cementation should be implemented. Subsequent experiments on the compaction behaviour of the produced samples could give important implications on the impact of early carbonate during burial.

## Supporting information

S1 Table Detailed information on the natural sand material used in the experiments. (DOCX)

S2 Table Operating conditions ICP-MS. CCT: Collison Cell Technology; LOD: Limit of Detection.
(DOCX)

S3 Table Calculations for ternary plots shown in Fig 4 from petrographic data of compositional data. (DOCX)

S4 Table Summary of measurements for grain-coating cement. (DOCX)
