# Supplementary material for: From loose sand to sandstone: An experimental approach on early calcite precipitation in sands of siliciclastic and mixed carbonate-siliciclastic composition
Source: PLoS One. 2024 Oct 23;19(10):e0312479. doi: 10.1371/journal.pone.0312479 (PMC11498678; doi:10.1371/journal.pone.0312479)
Supplement: S1 Table — (DOCX) [file pone.0312479.s001.docx]

S 1 Table. Detailed information on the natural sand material used in the experiments.

| **Sample** | SYN_1 | SYN_M | SYN_C | SYN_Lf | SYN_Lc |
| --- | --- | --- | --- | --- | --- |
| **Location** | Colombia (siliciclastic), Malangsgrunnen, N Norway (bioclasts) | Molasse basin, S Germany | Calabria, S Italy | Colombia | |
| **Age** | modern | modern | modern | modern | |
| **Depositional  environment** | fluvial, marine shelf | shore | shore | fluvial | |
