# Supplementary material for: From loose sand to sandstone: An experimental approach on early calcite precipitation in sands of siliciclastic and mixed carbonate-siliciclastic composition
Source: PLoS One. 2024 Oct 23;19(10):e0312479. doi: 10.1371/journal.pone.0312479 (PMC11498678; doi:10.1371/journal.pone.0312479)
Supplement: S2 Table — CCT: Collison Cell Technology; LOD: Limit of Detection. (DOCX) [file pone.0312479.s002.docx]

S 2 Table. Operating conditions ICP-MS. CCT: Collison Cell Technology; LOD: Limit of Detection.

| **Parameter** | **Specification** |
| --- | --- |
| Plasma power | 1550 W |
| Cool gas flow | 14.0 min^-1^ |
| Auxiliary gas flow | 0.65 L min^-1^ |
| Nebulizer gas flow | 1.03 L min^-1^ |
| CCT (KED mode) flow | 5.0 L min^-1^ |
| CCT gas | 8 % H_2_ in He |
| Sampler/Skimmer | Material: Nickel |
| Spray Chamber Temperature | 2.7°C |
| Dwell time | 10 ms (40 ms for Se, As) |
| Number of Sweeps | 70 |
| Sample flow | 0.4 ml min^-1^ |
| LOD typical | < 0.1 ppb |
