# Supplementary material for: From loose sand to sandstone: An experimental approach on early calcite precipitation in sands of siliciclastic and mixed carbonate-siliciclastic composition
Source: PLoS One. 2024 Oct 23;19(10):e0312479. doi: 10.1371/journal.pone.0312479 (PMC11498678; doi:10.1371/journal.pone.0312479)
Supplement: S3 Table — (DOCX) [file pone.0312479.s003.docx]

S 3 Table. Calculations for ternary plots shown in Fig 4 from petrographic data of compositional data.

| Ternary diagrams (%) | |  |  |  |  |
| --- | --- | --- | --- | --- | --- |
|  | SYN_1 | SYN_M | SYN_C | SYN_Lf | SYN_Lc |
| **QFCI** |  |  |  |  |  |
| Q | 37.4 | 84.8 | 38.2 | 31.4 | 33.3 |
| F | 36.9 | 14.0 | 61.6 | 68.6 | 66.7 |
| CI | 25.7 | 1.2 | 0.3 | 0.0 | 0.0 |
| **QFRf** |  |  |  |  |  |
| Q | 43.5 | 48.3 | 23.5 | 31.0 | 32.5 |
| F | 19.7 | 6.3 | 25.9 | 52.5 | 46.4 |
| Rf | 36.8 | 45.3 | 50.5 | 16.5 | 21.1 |
| **RmRgRs** |  |  |  |  |  |
| Rm | 11.4 | 87.5 | 74.3 | 16.5 | 21.0 |
| Rg | 27.8 | 3.7 | 25.0 | 83.5 | 79.0 |
| Rs | 60.8 | 8.8 | 0.68 | 0.0 | 0.0 |
