# Supplementary material for: From loose sand to sandstone: An experimental approach on early calcite precipitation in sands of siliciclastic and mixed carbonate-siliciclastic composition
Source: PLoS One. 2024 Oct 23;19(10):e0312479. doi: 10.1371/journal.pone.0312479 (PMC11498678; doi:10.1371/journal.pone.0312479)
Supplement: S4 Table — (DOCX) [file pone.0312479.s004.docx]

S 4 Table. Summary of measurements for grain-coating cement.

| **Grain** | **No. of grains** | **Frequency of  grain-coating cement (%)** | **Grain Coat**  **Coverage (%)** | **Thickness (µm)** |
| --- | --- | --- | --- | --- |
|  |  | Mean | | |
| Bioclasts | 42 | 91.3 | 69.5 | 40.1 |
| Feldspars | 84 | 71.6 | 85.1 | 33.8 |
| Quartz | 214 | 32.0 | 86.2 | 29.3 |
| Rock fragments | 119 | 29.0 | 80.5 | 26.6 |
| Altered grains | 36 | 26.8 | 88.2 | 32.5 |
| Mica | 8 | 18.1 | 85.0 | 14.0 |
| Carbonate grains | 3 | 8.8 | 73.3 | 30.0 |
|  |  | Median | | |
| Bioclasts | 42 | 91.3 | 69.5 | 40.1 |
| Feldspars | 84 | 75.7 | 83.9 | 32.5 |
| Quartz | 214 | 15.4 | 88.8 | 42.2 |
| Rock fragments | 119 | 12.6 | 85.3 | 39.8 |
| Altered grains | 36 | 3.4 | 94.3 | 36.1 |
| Mica | 8 | 18.1 | 85.0 | 12.8 |
| Carbonate grains | 3 | 8.8 | 73.3 | 30.0 |
